# Association Between Volleyball Participation and Knee Osteoarthritis in Community-Dwelling Adults: A Cross-Sectional Analysis of the Osteoarthritis Initiative

**DOI:** 10.3390/healthcare14131937

**Published:** 2026-07-01

**Authors:** Yaohui Yang, Hairui Zhang, Zhiyao Zhao, Fangzheng Zhou, Xiaoning Liu

**Affiliations:** Orthopaedic Medical Center, Second Hospital of Jilin University, Changchun 130041, China

**Keywords:** Osteoarthritis Initiative (OAI), volleyball, knee osteoarthritis, knee pain

## Abstract

**Objective**: This cross-sectional study aimed to examine the association between recreational volleyball participation and knee osteoarthritis (KOA) risk in a community-based population, focusing on participation frequency, cumulative exposure periods, and clinical outcomes. **Methods**: Utilizing data from the Osteoarthritis Initiative (OAI) cohort (*n* = 2539; ages 45–79), volleyball engagement was assessed via the Historical Physical Activity Survey Instrument across four age periods (12–18, 19–34, 35–49, ≥50). Participants were stratified into non-volleyball, low-frequency, and high-frequency groups. Outcomes included knee pain (WOMAC score ≥ 1), radiographic OA (ROA), and symptomatic OA (SOA). Logistic regression models adjusted for age, sex, BMI, and race were employed to evaluate the associations. **Results**: Volleyball participation reported across all four age periods was associated with higher odds of ROA (adjusted OR = 2.394, 95%CI: 1.247–4.596, *p* = 0.009). High-frequency participation alone, however, was not associated with knee pain, ROA, or SOA (all *p* > 0.05). No significant associations were observed between cumulative participation and knee pain or SOA, and no dose–response relationship was found for participation frequency. **Conclusions**: Recreational volleyball participation was not associated with higher prevalence of knee pain or symptomatic radiographic KOA, outcomes that may be more clinically relevant than radiographic findings alone. Although participation across all four life periods was associated with higher odds of ROA, this structural finding should not be overinterpreted because it was based on a small subgroup and a non-monotonic pattern in a cross-sectional analysis.

## 1. Introduction

Volleyball became an official Olympic sport in 1964, and since the 1980s, the number of participants has gradually increased. Today, volleyball is ranked among the top five most popular sports worldwide, with an estimated 200 million participants globally [1]. Compared with high-impact contact sports such as basketball and soccer, volleyball is generally associated with a relatively lower risk of acute injuries. However, the sport still poses a considerable risk of overuse injuries [2]. Movements such as jumping, forceful spiking, and landing exert significant stress on the knee joints of players [3,4]. Therefore, although volleyball offers health benefits, its potential risks should also be considered.

Osteoarthritis (OA) is a prevalent degenerative joint disease and one of the leading causes of disability worldwide, contributing to an increasing global health burden [5]. Epidemiological data indicate that approximately 10% of men and 18% of women over the age of 60 are affected by OA [6]. Among the various types of OA, knee osteoarthritis (KOA) is the most common form [7], characterized by the progressive degeneration and loss of articular cartilage. The primary clinical manifestations include activity-related knee pain, functional impairment, and joint swelling [8].

Previous studies have identified the ankle, distal upper extremities (wrist/hand/fingers), and knee as the most frequently injured anatomical sites in volleyball players [4]. Additionally, volleyball participation has been associated with an increased risk of osteophyte formation and meniscal pathology [9]. Nevertheless, most existing research has focused on professional adolescent or adult athletes, limiting the generalizability of findings to the broader population [10,11,12]. Volleyball participation patterns vary substantially by age, sex, and socioeconomic background, particularly across cohorts exposed to differing opportunities for physical education and organized sports. Understanding the association between lifetime volleyball participation and KOA-related outcomes may help clarify whether volleyball has joint-health implications distinct from other community sports. Therefore, our study intends to explore the potential association between volleyball participation and knee osteoarthritis in a community-based population. We hypothesized that cumulative volleyball participation across life stages would not be associated with a significantly higher risk of radiographic or symptomatic KOA compared with non-participants.

## 2. Methods

### 2.1. Osteoarthritis Initiative

The Osteoarthritis Initiative (OAI) is a multi-center, longitudinal, observational study sponsored by the National Institutes of Health (part of the Department of Health and Human Services). Between February 2004 and May 2006, the OAI enrolled 4796 participants aged 45 to 79 years, including individuals with symptomatic knee osteoarthritis and those at high risk of developing the condition. Participants were followed up annually, with over 90% retention during the initial 48 months. The study collected extensive clinical, radiographic, and biological data, including X-ray imaging, magnetic resonance imaging (MRI), and biospecimens such as blood, urine, and DNA. The OAI research team included institutions such as the University of Maryland School of Medicine, Ohio State University, the University of Pittsburgh, Rhode Island Hospital, and the University of California, San Francisco. This study received approval from each participating OAI site and the Baylor College of Medicine Institutional Review Board. Written informed consent was obtained from all participants before enrollment. Publicly available OAI data can be accessed at https://nda.nih.gov/oai/. We accessed the data on 1 June 2024. All OAI participants provided written informed consent, and the study was approved by the institutional review boards of all participating centers. The public-use dataset is de-identified; therefore, authors had no access to information that could identify individual participants during or after data analysis.

### 2.2. Lifetime Activity Assessment Tool

As part of the 96-month follow-up, the Historical Physical Activity Survey Instrument was used to collect self-reported physical activity data. Participants completed the survey between September 2012 and October 2014, recalling their past physical activity engagement. The questionnaire included 37 types of recreational sports, including volleyball. Participants were required to report whether they had participated in any activity at least 10 times, with each session lasting more than 20 min, across four distinct age periods: 12–18 years, 19–34 years, 35–49 years, and ≥50 years. Additionally, participants identified the top three most frequently performed recreational activities during each period to further assess long-term physical activity patterns. (Supplementary Table S1).

### 2.3. WOMAC Pain Assessment

The Western Ontario and McMaster Universities Osteoarthritis Index (WOMAC) is a self-reported health assessment tool used to evaluate knee pain, stiffness, and functional limitations, generating an overall score representing the participant’s knee health status. This study utilized the WOMAC pain score reported during baseline assessment, with a score range of 0 to 20 (0 indicates no pain, 20 indicates severe pain) [13,14].

### 2.4. Knee Radiographic Assessment

The OAI database contains semi-quantitative (SQ) radiographic knee data from baseline to 48-month follow-up, including the Kellgren-Lawrence (K-L) grading system. K-L grading was performed by trained radiologists based on serial X-ray images. If K-L data were only available for one knee during a given follow-up visit and the recorded K-L grade was <2, then data from the nearest follow-up visit were used instead. This study prioritized the use of bilateral, fixed-flexion, weight-bearing posterior–anterior (PA) knee X-rays from the 48-month follow-up visit (*n* = 2287) [15]. For 252 participants without 48-month K-L data, the closest available follow-up data were used: baseline (*n* = 12), 12-month (*n* = 44), 24-month (*n* = 53), and 36-month (*n* = 143).

### 2.5. Outcome Measures

**Figure 1:**
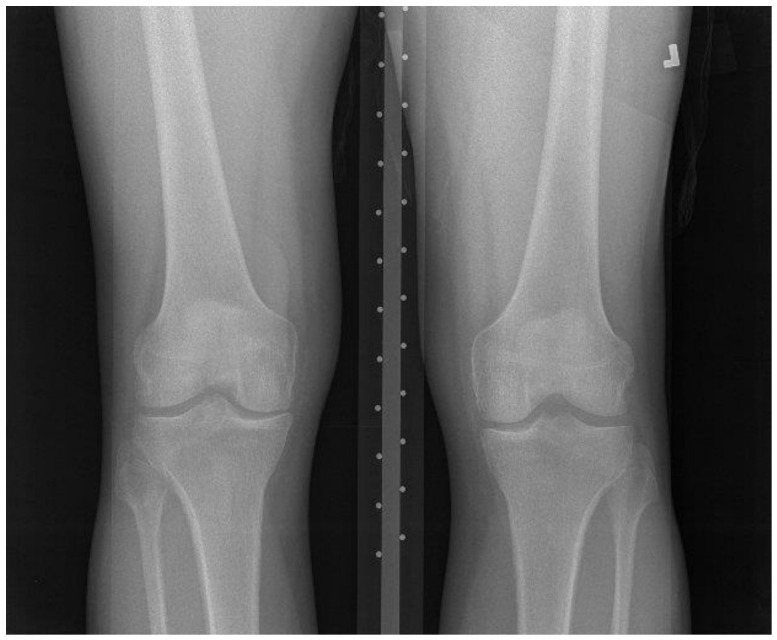
The knee X-ray image of a 65-year-old participant involved in volleyball across all four periods.

We defined a WOMAC pain score of 0 as the absence of knee pain, while a WOMAC pain score of 1–20 was classified as the presence of knee pain. Radiographic osteoarthritis (ROA) was defined as a Kellgren-Lawrence (K-L) grade of ≥2 in at least one knee. If an individual exhibited both knee pain (WOMAC ≥ 1) and ROA (K-L ≥ 2), they were classified as having symptomatic radiographic osteoarthritis (SOA). Demographic and clinical variables were systematically retrieved from the Osteoarthritis Initiative (OAI) database, encompassing age, racial/ethnic background, gender, BMI (body mass index), tobacco use history, and prior total knee arthroplasty (TKA) records. If a participant had undergone TKA in either knee, they were considered to have knee pain, ROA, and SOA. For example, a 65-year-old Caucasian male with a BMI of 30.9 kg/m^2^ has participated in volleyball across four periods and presents with knee pain. His X-ray shows significant narrowing of the left knee joint space, with a Kellgren-Lawrence (K-L) grade of 3 (Figure 1).

### 2.6. Volleyball Participants

Volleyball participation was determined using the HPASI-derived OAI lifetime physical activity variables. Individuals who reported volleyball participation in any age period (12–18 years, 19–34 years, 35–49 years, or 50 years or older) were classified as volleyball participants. Participants who listed volleyball among their top three recreational activities in at least one age period were categorized as high-frequency volleyball participants, whereas the remaining volleyball participants were classified as low-frequency volleyball participants. Thus, participants were divided into three groups: (A) non-volleyball participants, (B) low-frequency volleyball participants, and (C) high-frequency volleyball participants. For further analysis, volleyball participants were categorized according to the number of age periods in which volleyball participation was reported: one period, two periods, three periods, or all four periods. Participants classified as non-volleyball players may still have participated in other sports or physical activities; therefore, the comparison reflects volleyball-specific exposure rather than overall physical activity. The top-three activity definition should be interpreted as a relative indicator of activity prominence within a life period, not as a precise measure of weekly frequency, session duration, intensity, or cumulative knee loading.

### 2.7. Statistical Analysis

Intergroup comparisons of anthropometric parameters (age and BMI) were conducted using statistically validated independent samples *t*-test analysis. Pearson’s chi-square test was used to compare race, sex, smoking status, and TKA history. Logistic regression analysis was conducted to examine the association between volleyball participation frequency or periods and knee pain, ROA, and SOA. Model 1 was an unadjusted model. Model 2 was adjusted for age, sex, BMI, and race as covariates. Given the cross-sectional design, all results represent associations rather than causal effects. All statistical analyses were performed using IBM SPSS Statistics 25 (IBM Corp., Armonk, NY, USA), applying a significance threshold of α = 0.05 for inferential statistical determinations.

## 3. Results

**Figure 2:**
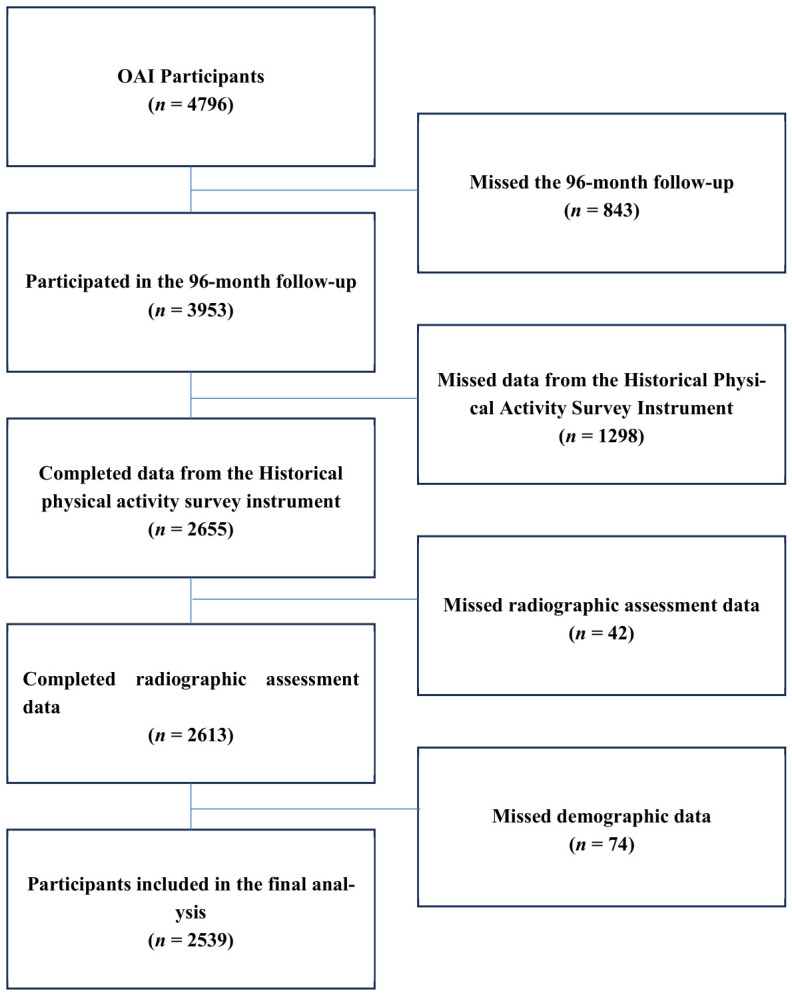
Flow diagram reflecting how 2539 participants were selected from the original 4796 Osteoarthritis Initiative participants. OAI: Osteoarthritis Initiative.

This study was based on the OAI database. After strict data screening criteria, 2257 individuals who did not meet the requirements were excluded, including those who missed the 96-month follow-up data, those with missing historical physical activity survey instrument data, those with missing radiographic data, and those with missing demographic data. The analytical cohort ultimately comprised 2539 eligible participants following rigorous screening protocols (Figure 2).

The analytical cohort (*n* = 2539) demonstrated a mean age of 64.4 ± 9.0 years, with gender distribution showing 1113 male participants (43.8%) and 1426 females (56.2%). There were 2012 (79.2%) White or Caucasian individuals and 464 (18.3%) Black or African American individuals. The average BMI recorded was 28.4 (SD, 4.7) kg/m^2^, and a total of 27 (1.1%) individuals had received TKA. The results of the Independent Samples *t*-test showed that volleyball participants had a younger average age and a marginally higher BMI. The results of Pearson’s Chi-squared Test showed that the proportion of males was higher among volleyball participants, but the proportion of non-volleyball participants who had received TKA was higher (Table 1).

Among the 1162 volleyball participants, the largest number, 428 (36.8%), participated in volleyball only at the age of 12–18. The second-largest number, 258 (22.2%), participated in volleyball both at the age of 12–18 and 19–34. The number of participants who participated in volleyball at the age of 19–34 and over fifty was the least, only 3 (0.3%) (Table 2).

Logistic regression analysis was used, with non-volleyball participants as the reference group, to explore the associations between volleyball participation frequency and knee pain, ROA, and SOA. Low-frequency and high-frequency volleyball participation were not significantly associated with knee pain, ROA, or SOA in either the crude or adjusted models. High-frequency participants showed slightly higher odds of knee pain and SOA than non-participants, but these associations did not reach statistical significance (Table 3).

We further evaluated the associations between the number of volleyball-participation periods and knee pain, ROA, and SOA. In the adjusted model, the association between participation periods and ROA was non-monotonic rather than dose-dependent. Compared with non-participants, participants who reported volleyball participation during one life period had lower odds of ROA (OR = 0.800, 95%CI: 0.655–0.977, *p* = 0.029), whereas those who participated across all four life periods had higher odds of ROA (OR = 2.394, 95% CI:1.247–4.596, *p* = 0.009). No statistically significant associations with ROA were observed for the two-period group (OR = 0.793, 95%CI:0.620–1.014, *p* = 0.064) or the three-period group (OR = 1.323, 95%CI:0.911–1.921, *p* = 0.141). Participation-period categories were not significantly associated with knee pain or SOA in the adjusted model (Table 4). This pattern did not support a simple cumulative exposure–response relationship.

## 4. Discussion

Our original hypothesis was not fully supported. In this community-dwelling OAI cohort, the top-three volleyball activity status was not significantly associated with knee pain, ROA, or SOA. However, the participation-period analysis showed a non-monotonic pattern for ROA: one-period participation was associated with lower odds of ROA, whereas participation across all four life periods was associated with higher odds of ROA. The lower odds observed in the one-period group should not be interpreted as evidence of a protective effect. Similarly, although the four-period group showed higher odds of ROA, this subgroup included only 53 participants, and the estimate may therefore be unstable and vulnerable to chance variation. These findings may be influenced by residual confounding, exposure misclassification, recall bias, multiplicity, and the cross-sectional design. Therefore, they should be interpreted as exploratory associations rather than causal effects. Accordingly, the present analysis cannot determine whether volleyball participation itself has an independent effect on the development of knee osteoarthritis.

One possible explanation for the four-period ROA finding is long-term knee loading related to repeated jumping, landing, and rotational movements in volleyball; however, the non-monotonic pattern observed in the period-based analysis argues against a simple cumulative exposure–response interpretation. Volleyball involves repeated jumping, landing, and rotational movements, which may increase mechanical stress on articular cartilage, menisci, and subchondral bone over time. Prior imaging studies in volleyball athletes have reported osteophytes and meniscal abnormalities, supporting the possibility that long-term sport-specific loading may influence knee joint morphology. However, this interpretation remains speculative because detailed information on sport-specific injury history, playing intensity, competitive level, playing position, and cumulative mechanical loading was unavailable.

Notably, despite the association with ROA, volleyball participation was not associated with knee pain or symptomatic OA. This dissociation between structural abnormalities and symptoms has been widely reported in OA research and suggests several possible explanations. Previous population-based and systematic-review evidence has shown substantial discordance between radiographic KOA and knee pain, indicating that radiographic findings alone are an imprecise marker of clinically meaningful symptoms or disability [16,17]. In addition, OA-related pain is influenced not only by radiographic structural change but also by synovitis, bone marrow lesions, peripheral and central pain sensitization, psychological factors, and activity level [18]. Therefore, the higher odds of ROA observed in the four-period group may reflect subclinical structural adaptation or early degenerative change rather than established symptomatic disease. This distinction is important because radiographic findings alone should not be interpreted as evidence of clinically relevant knee impairment.

Most previous studies on volleyball-related knee outcomes have focused on professional or elite athletes [9,10,19]. Using community-based OAI data and a lifetime physical activity instrument, the present study provides a complementary perspective on recreational volleyball participation among adults. Prior OAI-based studies have also shown that the relationship between recreational physical activity and KOA varies by activity type, with some activities showing neutral or potentially favorable associations and others showing less favorable associations [20,21,22,23,24,25]. These findings highlight the need to evaluate specific recreational activities separately rather than assuming uniform effects of all sports on knee health.

This study has several strengths. It utilized data from the OAI, one of the largest and most rigorously characterized osteoarthritis cohorts worldwide, allowing adjustment for important covariates such as age, BMI, sex, and race. Volleyball participation was assessed using a structured lifetime physical activity instrument, enabling evaluation of exposure patterns across distinct developmental stages. The inclusion of community-dwelling adults, rather than only professional athletes, allows evaluation of recreational volleyball participation outside elite sports settings.

Several limitations should be acknowledged. First, this study was cross-sectional and cannot establish temporal or causal relationships. Lifetime physical activity was recalled at the 96-month visit, whereas radiographic outcomes were obtained mainly from the 48-month visit or the nearest available follow-up. Therefore, some recalled activity may not have clearly preceded radiographic assessment, and the temporal ordering between volleyball exposure and ROA cannot be fully determined. Second, the HPASI captured whether volleyball participation met a minimum threshold and whether volleyball was listed among the top three activities within each life period, but it did not quantify weekly hours, session duration, playing position, training intensity, competitive level, or cumulative knee loading. Therefore, exposure misclassification and recall bias are possible. In particular, the top-three activity definition should be interpreted as an indicator of activity prominence within a given life period rather than a precise measure of true frequency, intensity, or cumulative mechanical exposure. Third, although the adjusted model included age, sex, BMI, and race, several potentially important confounders could not be fully incorporated because of unavailable, incomplete, or missing data. These included prior anterior cruciate ligament injury, meniscal injury, other knee trauma, and sport-specific injury history. Prior ligament injury or degeneration may contribute to KOA through interactions among ligament damage, cartilage degeneration, and subchondral bone changes [26]. Other sports participation, occupational knee loading, and lower-limb malalignment were also not fully captured; notably, valgus malalignment has been reported to influence the incidence and progression of KOA [27]. As a result, residual confounding remains possible and may have affected both the direction and magnitude of the observed associations; therefore, these associations should not be interpreted as evidence that volleyball participation itself independently causes radiographic KOA. Another related concern is the possibility of a healthy participant effect. Individuals who were able to continue volleyball participation across multiple life periods may have differed systematically from non-participants in health status, physical function, health-related behaviors, socioeconomic background, or access to recreational opportunities. These differences were not fully captured by the available covariates and may have contributed to residual confounding. Fourth, participants with prior TKA were classified as positive for knee pain, ROA, and SOA, and TKA was more common among non-volleyball participants. This definition may have influenced the outcome distribution toward the non-volleyball group. In addition, the use of K-L grade ≥ 2 to define ROA may not capture all clinically relevant KOA phenotypes and may introduce outcome misclassification. Finally, multiple outcomes, exposure categories, and regression models were examined, and no formal correction for multiple comparisons was applied. Therefore, chance findings due to multiplicity cannot be excluded. The four-period subgroup was also small, which may reduce the stability of the estimate. The OAI cohort was not population-based and included individuals with existing symptomatic KOA or elevated OA risk, limiting the generalizability of the findings.

Future studies should use prospective cohort designs with more detailed volleyball-specific exposure assessment, including weekly frequency, session duration, playing position, training intensity, competitive level, and injury history. More precise OA phenotyping is also needed, because KOA includes multiple structural phenotypes, such as patellofemoral OA and tibiofemoral OA, which may have distinct risk factors and disease mechanisms [28,29]. In addition, repeated radiographic and MRI assessments could help clarify whether prolonged volleyball participation is associated with soft-tissue injury, early structural adaptation, or progressive degenerative change [30]. In summary, recreational volleyball participation was not associated with knee pain or symptomatic radiographic OA in this cross-sectional analysis. However, the higher odds of ROA among participants reporting volleyball participation across all four life periods suggest that the potential structural implications of prolonged lifelong participation should be interpreted cautiously and evaluated in future longitudinal studies.

## 5. Conclusions

In conclusion, this exploratory cross-sectional analysis of the OAI analytic sample found no association between retrospectively reported recreational volleyball participation and a higher prevalence of knee pain or symptomatic radiographic KOA. These clinically relevant symptomatic outcomes may be more directly meaningful to patients than radiographic findings alone. Participation across all four life periods was associated with higher odds of ROA, but this finding arose from a small subgroup and a non-monotonic period-based pattern. Given the retrospective exposure assessment, incomplete injury history, residual confounding, temporal mismatch, multiplicity, and cross-sectional design, the present study cannot support firm conclusions about a causal relationship between volleyball participation and knee osteoarthritis. These findings should therefore be interpreted as descriptive and hypothesis-generating, and they require confirmation in prospective studies with detailed volleyball-specific exposure, injury, and longitudinal structural and symptomatic outcome data.

## Figures and Tables

**Table 1:** Characteristics of volleyball participants, non-volleyball participants, and all participants.

	Non-Volleyball Participants (*n* = 1377)	Volleyball Participants (*n* = 1162)	All Participants (*n* = 2539)	*p* Value
Participant Characteristics				
Age (yr)	65.6 ± 9.1	62.9 ± 8.6	64.4 ± 9.0	<0.001 ^a^
Gender				0.003 ^b^
Male (%)	567 (41.2)	546 (47.0%)	1113 (43.8%)	
Female (%)	810 (58.8%)	616 (53.0%)	1426 (56.2%)	
Race				0.344 ^b^
White or Caucasian (%)	1100 (79.9%)	912 (78.5%)	2012 (79.2%)	
Black or African American (%)	239 (17.4%)	225 (19.4%)	464 (18.3%)	
BMI (kg/m^2^)	28.2 ± 4.7	28.6 ± 4.8	28.4 ± 4.7	0.046 ^a^
Smoke (%)	638 (46.3%)	508 (43.7%)	1146 (45.1%)	0.187 ^b^
TKA (%)	20 (1.5%)	7 (0.6%)	27 (1.1%)	0.037 ^b^

Note—Continuous data are presented as means ± SDs; Categorical data are presented as number of participants, with percentages in parentheses. BMI: Body Mass Index, TKA: Total knee arthroplasty. ^a^ Independent Samples *t*-test. ^b^ Pearson’s Chi-squared Test.

**Table 2:** The specific period of participation in volleyball *.

Ages 12–18	Ages 19–34	Ages 35–49	Ages ≥ 50	Frequency (Percentage)
X				428 (36.8%)
X	X			258 (22.2%)
	X			138 (11.9%)
X	X	X		116 (10.0%)
	X	X		64 (5.5%)
X	X	X	X	53 (4.6%)
		X		30 (2.6%)
	X	X	X	20 (1.7%)
			X	16 (1.4%)
X		X		11 (0.9%)
		X	X	10 (0.9%)
X			X	7 (0.6%)
X	X		X	4 (0.3%)
X		X	X	4 (0.3%)
	X		X	3 (0.3%)
				1162 (100%)

* X represents the participant’s involvement in volleyball during that period.

**Table 3:** Odds Ratio of knee pain, ROA, SOA for low-frequency and high-frequency participants.

Frequency of Participating in Volleyball	Model 1		Model 2	
Outcome: Knee Pain	OR (95%CI)	*p* value	OR (95%CI)	*p* value
Never Participated (*n* = 1377)	Referent		Referent	
Low Frequency (*n* = 881)	1.055 (0.887, 1.263)	0.558	1.038 (0.812, 1.250)	0.693
High Frequency (*n* = 281)	1.196 (0.905, 1.580)	0.209	1.108 (0.830, 1.478)	0.487
Outcome: ROA	OR (95%CI)	*p* value	OR (95%CI)	*p* value
Never Participated (*n* = 1377)	Referent		Referent	
Low Frequency (*n* = 881)	0.912 (0.769, 1.082)	0.290	0.871 (0.729, 1.041)	0.130
High Frequency (*n* = 281)	0.991 (0.764, 1.284)	0.944	0.936 (0.713, 1.228)	0.633
Outcome: SOA	OR (95%CI)	*p* value	OR (95%CI)	*p* value
Never Participated (*n* = 1377)	Referent		Referent	
Low Frequency (*n* = 881)	0.998 (0.875, 1.207)	0.987	0.947 (0.776, 1.155)	0.590
High Frequency (*n* = 281)	1.086 (0.817, 1.443)	0.569	1.026 (0.762, 1.380)	0.866

Note—OR: Odds Ratio; CI: Confidence interval; Model 1 was unadjusted; Model 2 was adjusted for age, sex, BMI, and race.

**Table 4:** Odds Ratio of knee pain, ROA, SOA based on the number of periods of volleyball participation.

Number of Periods	Model 1		Model 2	
Outcome: Knee Pain	OR (95%CI)	*p* value	OR (95%CI)	*p* value
Never Participated (*n* = 1377)	Referent		Referent	
One period (*n* = 612)	1.090 (0.890, 1.336)	0.405	1.045 (0.847, 1.289)	0.683
Two periods (*n* = 353)	1.042 (0.812, 1.336)	0.747	1.034 (0.800, 1.337)	0.799
Three periods (*n* = 144)	1.198 (0.824, 1.741)	0.345	1.129 (0.767, 1.662)	0.539
Four periods (*n* = 53)	1.080 (0.600, 1.943)	0.798	1.116 (0.611, 2.040)	0.721
Outcome: ROA	OR (95%CI)	*p* value	OR (95%CI)	*p* value
Never Participated (*n* = 1377)	Referent		Referent	
One period (*n* = 612)	0.840 (0.694, 1.018)	0.075	0.800 (0.655, 0.977)	***0.029***
Two periods (*n* = 353)	0.848 (0.671, 1.073)	0.170	0.793 (0.620, 1.014)	0.064
Three periods (*n* = 144)	1.368 (0.955, 1.960)	0.087	1.323 (0.911, 1.921)	0.141
Four periods (*n* = 53)	2.239 (1.187, 4.225)	***0.013***	2.394 (1.247, 4.596)	***0.009***
Outcome: SOA	OR (95%CI)	*p* value	OR (95%CI)	*p* value
Never Participated (*n* = 1377)	Referent		Referent	
One period (*n* = 612)	0.902 (0.725, 1.121)	0.352	0.857 (0.683, 1.076)	0.184
Two periods (*n* = 353)	1.075 (0.829, 1.393)	0.586	1.009 (0.769, 1.323)	0.949
Three periods (*n* = 144)	1.299 (0.899, 1.878)	0.163	1.220 (0.830, 1.792)	0.312
Four periods (*n* = 53)	1.379 (0.772, 2.465)	0.278	1.406 (0.768, 2.574)	0.269

Note—OR: Odds Ratio; CI: Confidence interval; Model 1 was unadjusted; Model 2 was adjusted for age, sex, BMI, and race. Bold italic *p* values indicate statistical significance (*p* < 0.05).

## Data Availability

The datasets generated and analysed during the current study are available in the OAI (https://nda.nih.gov/oai).

## Supplementary Materials

The following supporting information can be downloaded at https://www.mdpi.com/article/10.3390/healthcare14131937/s1, Table S1. Summary of OAI lifetime physical activity items.

## Abbreviations

Knee osteoarthritis (KOA), the Osteoarthritis Initiative (OAI), Osteoarthritis (OA), magnetic resonance imaging (MRI), The Western Ontario and McMaster Universities Osteoarthritis Index (WOMAC), semi-quantitative (SQ), Kellgren-Lawrence (K-L), posterior–anterior (PA), BMI (body mass index), total knee arthroplasty (TKA), Odds Ratio (OR).
