# Supplementary material for: Association Between Volleyball Participation and Knee Osteoarthritis in Community-Dwelling Adults: A Cross-Sectional Analysis of the Osteoarthritis Initiative
Source: Healthcare (Basel). 2026 Jul 1;14(13):1937. doi: 10.3390/healthcare14131937 (PMC13361759; doi:10.3390/healthcare14131937)
Supplement: Supplementary file 1 [file healthcare-14-01937-s001.zip › healthcare-4358579-supplementary.pdf]

Supplementary Table S1. Summary of OAI lifetime physical activity items.

Item	OAI documentation contains
Survey source	The OAI 96-month self-administered exercise questionnaire includes lifetime recreational physical activity information.
Exercise activity list	The exercise section includes 37 recreational activities: aerobic dance or step aerobics; badminton; baseball or softball; basketball; bicycling or spinning; bowling; canoeing, rowing or kayaking; carpentry; dancing; elliptical trainer; fishing; football; gardening or yardwork; golfing with a cart; golfing without a cart; gymnastics; handball; hockey; ice skating; jogging or running; jumping rope; racquetball or squash; roller blading or skating; sailing; snow skiing, cross-country or Nordic track; snow skiing, downhill; soccer; Stairmaster or walking up and down stairs; strength or weight training; swimming; table tennis or Ping Pong; Tai Chi; tennis, doubles; tennis, singles; volleyball; water aerobics; and yoga.
Age periods	Participation was recorded separately for four age periods: 12-18 years, 19-34 years, 35-49 years, and 50 years and older.
Basic volleyball participation	For each age period, the questionnaire records whether a participant reported volleyball participation.
Top three activities	For each age period, the questionnaire also records the participant’s top three recreational activities.
Additional activity information	For top-three activities, the OAI documentation includes information such as years of participation, months per year, times per month, and whether the activity was performed competitively.
Interpretation of exposure	The activity information is retrospective and self-reported.

Note. OAI, Osteoarthritis Initiative; HPASI, Historical Physical Activity Survey Instrument.
